# Development and Validation of a Nomogram Prognostic Model for Resected Limited-Stage Small Cell Lung Cancer Patients

**DOI:** 10.1245/s10434-020-09552-w

**Published:** 2021-03-02

**Authors:** Qingpeng Zeng, Jiagen Li, Fengwei Tan, Nan Sun, Yousheng Mao, Yushun Gao, Qi Xue, Shugeng Gao, Jun Zhao, Jie He

**Affiliations:** Department of Thoracic Surgery, National Cancer Center/National Clinical Research Center for Cancer/Cancer Hospital, Chinese Academy of Medical Sciences and Peking Union Medical College, No. 17, Panjiayuan Nanli, Beijing, 100021 China

## Abstract

**Background:**

In this study, we developed and validated nomograms for predicting the survival in surgically resected limited-stage small cell lung cancer (SCLC) patients.

**Methods:**

The SCLC patients extracted from the Surveillance, Epidemiology, and End Results database between 2000 and 2014 were reviewed. Significant prognostic factors were identified and integrated to develop the nomogram using multivariable Cox regression. The model was then validated internally by bootstrap resampling, and externally using an independent SCLC cohort diagnosed between 2000 and 2015 at our institution. The prognostic performance was measured by the concordance index (C-index) and calibration curve.

**Results:**

A total of 1006 resected limited-stage SCLC patients were included in the training cohort. Overall, 444 cases from our institution constituted the validation cohort. Seven prognostic factors were identified and entered into the nomogram construction. The C-indexes of this model in the training cohort were 0.723, 0.722, and 0.746 for predicting 1-, 3-, and 5-year overall survival (OS), respectively, and 0.816, 0.710, and 0.693, respectively, in the validation cohort. The calibration curve showed optimal agreement between nomogram-predicted survival and actual observed survival. Additionally, significant distinctions in survival curves between different risk groups stratified by prognostic scores were also observed. The proposed nomogram was then deployed into a website server for convenient application.

**Conclusions:**

We developed and validated novel nomograms for individual prediction of survival for resected limited-stage SCLC patients. These models perform better than the previously widely used staging system and may offer clinicians instructions for strategy making and the design of clinical trials.

**Supplementary Information:**

The online version of this article (10.1245/s10434-020-09552-w) contains supplementary material, which is available to authorized users.

Lung cancer still remains the leading cause of cancer-related deaths worldwide. Small cell lung cancer (SCLC) accounts for approximately 15–20% of lung cancer patients, of whom approximately 30% are non-metastatic at initial diagnosis.^1^ SCLC is characterized by rapid progression, high aggressiveness, and inferior prognosis; multimodality therapy, including chemotherapy and radiotherapy, is still the standard management of this disease.^2^ The role of surgery in the treatment of SCLC is currently considered very limited since the two previous clinical trials^3^^,^^4^ showed no survival benefit from the introduction of surgery into the treatment modality; however, several studies have revealed that surgery may achieve favorable survival outcomes in patients with early-stage disease.^5^^,^^6^ Currently, the National Comprehensive Cancer Network (NCCN) guidelines recommend surgery for selected cases of clinical stage *T*1–2, *N*0 SCLC.^7^

The two-tier staging system (limited disease and extensive disease) introduced by the Veterans Administration Lung Study Group (VALSG) was used as the foundation of the treatment strategy and the major prognostic parameter; however, individual survival differs widely in the same stage. The American Joint Committee on Cancer (AJCC) 7th TNM staging system was reported to contribute to a more precise prognosis and has been adopted for the staging of SCLC.^8^^,^^9^ In addition, several previous studies have revealed other independent prognostic factors, including sex, lobectomy, adjuvant chemotherapy, or radiotherapy, for surgically treated SCLC.^10^^–^12 Hence, based on these above factors, a more individualized prediction of survival could be achieved. Nomogram models have been widely used as a feasible tool to predict individualized prognosis for cancer patients, which could benefit treatment strategy making and clinical trials.

To date, four nomogram studies regarding SCLC have been published,^13^^–^16 however all the studies include all-staged SCLC patients and did not analyze patients who underwent surgery. Thus, we aimed to establish a nomogram to predict survival outcomes after surgery in limited-stage SCLC using a large cohort from the Surveillance, Epidemiology, and End Results (SEER) database. In addition, this nomogram model was externally validated by a separate cohort from the Cancer Institute and Hospital of the Chinese Academy of Medical Sciences (CICAMS).

## Patients and Methods

### Study Population

The SEER is a population-based database that covers approximately 28% of the US population. The latest SEER data, released in April 2019, includes cancer incidence data ranging from January 1975 to December 2016. A total of 94,247 SCLC cases were identified from the database using SEER* Stat version 8.3.5 (National Cancer Institute, Bethesda, MD, USA), of which 1006 resected limited-stage cases met our inclusion criteria and entered the training cohort. The specific criteria and codes for inclusion or exclusion are shown in electronic supplementary Fig. S1.

To examine the generalizability of this model, an external validation cohort was constructed from the CICAMS. We reviewed our database of patients with histologically confirmed SCLC from January 2000 to December 2015. A total of 444 consecutive resected limited-stage SCLC patients were identified. Laboratory tests, pulmonary function test, computed tomography of the chest and upper abdomen, bronchoscopy, brain magnetic resonance imaging, emission computed tomography bone scans, or positron emission tomography of the whole body were routinely performed prior to surgery at our institution. Clinical data were retrieved from the medical record database and survival information was obtained from our follow-up center or by contacting the patients. Ethical approval was given by the Research Ethics Committee of CICAMS, which waived the requirement for informed patient consent because of the retrospective nature of this study.

### Variables

For each patient, several variables were gathered from the SEER database, i.e. age, sex, race, tumor location, surgery, number of lymph nodes dissected (LND), number of lymph node metastases (LNM), histology type, stage, additional treatment (chemotherapy or radiotherapy), survival months, causes of death, and vital status. For the validation cohort, the same variables were also extracted. In terms of surgery, surgical codes indicating the resection of fewer than one lobe (wedge resection, segmental resection) were categorized as sublobectomy. The combined histology subtype refers to SCLC accompanied by other components (such as adenocarcinoma or squamous carcinoma). In addition, we revised the TNM categories according to the Collaborative Staging Manual and Coding Instructions for the AJCC 8th staging system.^17^ We assembled the IA1, IA2, IA3 stages as IA disease as no significant difference in survival was found among these substages.^18^ According to the SEER summary staging system, we further divided VALSG limited disease into two subgroups: localized disease (tumor confined to the primary organ without LNM) and regional disease (tumor invaded directly to the adjacent organ/tissue or regional LNM). Information regarding chemotherapy or radiotherapy was also included, as additional therapy; however, we were unable to define neoadjuvant or adjuvant therapy due to the lack of sequence of the additional treatment. In the SEER database, regretfully, variable of visceral pleural invasion for lung cancer was unavailable before 2010, information regarding prophylactic cranial radiation was missing during this period, and more than one-third of the differentiation grade were undefined, hence we did not include these parameters in the analysis.

### Construction of the Nomogram

The nomogram was developed using a training cohort of 1006 patients. Variables entered into the final analysis included age, sex, race, laterality, primary site, surgery, LND, LNM, histology type, TNM stage, chemotherapy, and radiotherapy. Overall survival (OS) was calculated according to vital status, and censored subjects were recorded based on the status of ‘alive’, for OS. Significant prognostic correlating variables were analyzed using the univariate Cox proportional hazards regression model and the Wald test. Variables with a *p* value < 0.05 entered the multivariate Cox regression analysis for eliminating redundant variables via the backward stepwise process based on Akaike information criterion.^19^ The prognostic nomogram was constructed based on the risk score calculated by the final Cox regression model.

### Model Performance and Validation

The performance for predicting survival of this nomogram model was evaluated using the concordance index (C-index), which represents a concordance measure analogous to the area under the receiver operating characteristic (ROC). The C-index ranges from 0.5 (indicating no better than random chance) to 1.0 (indicating perfect prediction).^20^ Calibration curves of the nomogram for 1-, 3-, and 5-year survival were plotted to evaluate the consistency between predicted survival probability and actual survival proportion. A perfectly calibrated model would present with a 45-degree curve. For model validation, 1000 bootstrap resamples in the training cohort were applied for internal validation. Furthermore, an independent external validation was conducted using the CICAMS cohort. The two conventional staging models—AJCC 8th TNM staging system and VALSG staging system—were also assessed for the prognostic performance, in both the training and validation cohorts. For the present study, the VALSG system incorporated modified localized disease and regional disease. In addition, the area under the curve (AUC) of the time-dependent ROC was calculated each month, from months 1 to 60. The decision curve analysis (DCA) was also conducted to evaluate the benefits and advantages of our new predicting model over the other two staging models.

For assessing the discriminate ability of the model, we also grouped patients into several risk subsets according to prognostic scores in the training cohort. The cut-off values were defined using the X-tile software version 3.6.1 (Yale University School of Medicine, New Haven, CT, USA), which could recognize the optimal cut-off values for continuous variables through calculating the largest Chi square and minimum *p* values. These cut-off values were then applied to the different TNM categories and the validation cohort; the respective log-rank *p* values were calculated to compare the difference in survival.

### Statistical Analysis

Statistical analysis was performed using SPSS 23.0 (IBM Corporation, Armonk, NY, USA) and R version 3.6.1 (The R Foundation for Statistical Computing, Vienna, Austria). The R packages ‘survival’ (version 2.44-1.1), ‘foreign’ (version 0.8-72), ‘rms’ (version 5.1-3.1), ‘survivminer’ (version 0.4.5), and ‘timeROC’ (version 1.0.3) were used for nomogram construction and evaluation. Furthermore, the R packages ‘DynNom’ (version 5.0.1) and ‘rsconnect’ (version 0.8.16) were applied for developing a user-friendly web-based interface for our nomogram. Kaplan–Meier survival analysis was used to assess distinctions in prognosis with a log-rank *p* value. A two-tailed *p* value < 0.05 was considered to be statistically significant.

## Results

### Characteristics of the Training and Validation Cohorts

The training cohort comprised 1006 patients with resected primary limited-stage SCLC, from the SEER database. There were 755 deaths over a median follow-up duration of 101.0 months (range 1–203 months). The validation cohort consisted of 444 cases of limited-stage SCLC and 222 deaths were observed over a median follow-up duration of 80.0 months (range 2 days to 213 months). Detailed demographic characteristics of patients in the training and validation cohorts are shown in Table 1. The median age at diagnosis was 63.5 years (range 34–90 years) and 50.5 years (range 19–82 years) in the training and validation cohorts, respectively. Both groups were predominant in the male sex. Lobectomy accounted for the major procedure in all enrolled cases. Over 10% of cases were diagnosed with combined SCLC in both groups.

**Table 1:** Demographic characteristics of the training and validation cohorts

Demographic characteristics	Overall	Training cohort	Validation cohort
No. of patients	1450	1006	444
Age, years			
< 60	508 (35.0)	261 (25.9)	247 (55.6)
60–70	542 (37.4)	399 (39.7)	143 (32.2)
> 70	400 (27.6)	346 (34.4)	54 (12.2)
Sex			
Female	647 (44.6)	534 (53.1)	113 (25.5)
Male	803 (55.4)	472 (46.9)	331 (74.5)
Race			
White	910 (62.8)	910 (90.5)	–
Black	73 (5.0)	73 (7.3)	–
Other	23 (1.6)	23 (2.3)	–
Laterality			
Left	668 (46.1)	451 (44.8)	217 (48.9)
Right	782 (53.9)	555 (55.2)	227 (51.1)
Primary site			
Upper lobe	773 (53.3)	582 (57.9)	191 (43.0)
Middle lobe	87 (6.0)	60 (6.0)	27 (6.1)
Lower lobe	533 (36.8)	307 (30.5)	226 (50.9)
Unknown	57 (3.9)	57 (5.7)	0 (0.0)
Surgery			
Lobectomy	1022 (70.5)	622 (61.8)	400 (90.1)
Sublobectomy	356 (24.6)	347 (34.5)	9 (2.0)
Pneumonectomy	72 (5.0)	37 (3.7)	35 (7.9)
Histology			
Pure SCLC	1231 (84.9)	834 (82.9)	397 (89.4)
Combined SCLC	219 (15.1)	172 (17.1)	47 (10.6)
LND group			
0–5	546 (37.7)	531 (52.8)	15 (3.4)
6–10	282 (19.4)	238 (23.7)	44 (9.9)
11–20	346 (23.9)	171 (17.0)	175 (39.4)
21–30	170 (11.7)	44 (4.4)	126 (28.4)
> 30	106 (7.3)	22 (2.2)	84 (18.9)
LNM group			
0	637 (43.9)	460 (45.7)	177 (39.9)
1–3	418 (28.8)	279 (27.7)	139 (31.3)
4–6	107 (7.4)	44 (4.4)	63 (14.2)
7–9	48 (3.3)	21 (2.1)	27 (6.1)
≥ 10	42 (2.9)	8 (0.8)	34 (7.7)
NO*	198 (13.7)	194 (19.3)	4 (0.9)
AJCC stage			
IA	377 (26.0)	303 (30.1)	74 (16.7)
IB	204 (14.1)	142 (14.1)	62 (14.0)
IIA	63 (4.3)	39 (3.9)	24 (5.4)
IIB	325 (22.4)	211 (21.0)	114 (25.7)
IIIA	375 (25.9)	237 (23.6)	138 (31.1)
IIIB	106 (7.3)	74 (7.4)	32 (7.2)
*T* stage			
*T*1a	98 (6.8)	76 (7.6)	22 (5.0)
*T*1b	284 (19.6)	226 (22.5)	58 (13.1)
*T*1c	228 (15.7)	159 (15.8)	69 (15.5)
*T*2a	402 (27.7)	265 (26.3)	137 (30.9)
*T*2b	140 (9.7)	65 (6.5)	75 (16.9)
*T*3	174 (12.0)	112 (11.1)	62 (14.0)
*T*4	124 (8.6)	103 (10.2)	21 (4.7)
*N* stage			
*N*0	761 (52.5)	580 (57.7)	181 (40.8)
*N*1	332 (22.9)	204 (20.3)	128 (28.8)
*N*2	357 (24.6)	222 (22.1)	135 (30.4)
VALSG stage			
Localized	658 (45.4)	488 (48.5)	170 (38.3)
Regional	792 (54.6)	518 (51.5)	274 (61.7)
Chemotherapy			
No/unknown	367 (25.3)	333 (33.1)	34 (7.7)
Yes	1083 (74.7)	673 (66.9)	410 (92.3)
Radiotherapy			
No/unknown	932 (64.3)	608 (60.4)	324 (73.0)
Yes	518 (35.7)	398 (39.6)	120 (27.0)

Data are expressed as *n* (%)

*SCLC* small cell lung cancer, *LND* lymph node dissected, *LNM* lymph node metastasis, NO^*^ no lymph nodes dissected, *AJCC* American Joint Committee on Cancer, *VALSG* Veterans Administration Lung Study Group

### Independent Prognostic Factors in the Training Cohort

Cox proportional hazards models were performed to assess the independent prognostic factors in the training cohort and the results are shown in Table 2. In univariate analysis, age, sex, surgery, *T* stage, *N* stage, LND, LNM, and chemotherapy were revealed to be significant correlating variables for OS. The univariate analysis survival curves are shown in electronic supplementary Fig. S2. N stage was not an independent variable for LNM and was hence excluded from the multivariate analysis. After multivariate analysis, age, sex, surgery, T stage, LND, LNM, and chemotherapy were demonstrated to be independent prognostic factors.

**Table 2:** Results of univariable and multivariate Cox proportional hazards regression analysis for overall survival

Variables	Univariable analysis		Multivariate analysis	
	HR (95% CI)	*p* Value	HR (95% CI)	*p* Value
Age, years				
< 60	1		1	
60–70	1.369 (1.133–1.654)	0.001	1.545 (1.273–1.876)	< 0.001
> 70	1.918 (1.585–2.322)	< 0.001	2.057 (1.686–2.510)	< 0.001
Sex				
Female	1		1	
Male	1.302 (1.128–1.502)	< 0.001	1.324 (1.145–1.532)	< 0.001
Race				
White	1			
Black	0.770 (0.576–1.029)	0.077		
Other	0.819 (0.506–1.325)	0.415		
Laterality				
Left	1			
Right	1.021 (0.885–1.179)	0.772		
Primary site				
Upper lobe	1			
Middle lobe	0.939 (0.687–1.285)	0.695		
Lower lobe	1.059 (0.902–1.243)	0.482		
Unknown	1.338 (0.993–1.803)	0.056		
Surgery				
Lobectomy	1		1	
Sublobectomy	1.506 (1.297–1.749)	< 0.001	1.313 (1.092–1.579)	0.004
Pneumonectomy	1.444 (0.997–2.091)	0.052	1.294 (0.882–1.898)	0.187
Histology				
Pure SCLC	1			
Combined SCLC	0.987 (0.815–1.195)	0.890		
*T* stage				
*T*1a	1		1	
*T*1b	1.282 (0.925–1.775)	0.136	1.263 (0.909–1.754)	0.165
*T*1c	1.382 (0.985–1.941)	0.062	1.341 (0.951–1.892)	0.094
*T*2a	1.560 (1.136–2.143)	0.006	1.621 (1.174–2.238)	0.003
*T*2b	1.427 (0.956–2.130)	0.082	1.737 (1.146–2.635)	0.009
*T*3	2.020 (1.423–2.868)	< 0.001	1.928 (1.345–2.764)	< 0.001
*T*4	2.301 (1.614–3.281)	< 0.001	2.051 (1.432–2.938)	< 0.001
*N* stage				
*N*0	1		NA	NA
*N*1	1.525 (1.275–1.825)	< 0.001		
*N*2	1.852 (1.555–2.206)	< 0.001		
LND				
0–5	1		1	
6–10	0.754 (0.631–0.901)	0.002	0.838 (0.681–1.032)	0.096
11–20	0.709 (0.577–0.871)	0.001	0.691 (0.543–0.879)	0.003
21–30	0.759 (0.527–1.092)	0.137	0.650 (0.437–0.967)	0.034
>30	0.542 (0.318–0.926)	0.025	0.525 (0.301–0.915)	0.023
LNM				
0	1		1	
1–3	1.800 (1.515–2.138)	< 0.001	1.813 (1.510–2.176)	< 0.001
4–6	2.200 (1.567–3.089)	< 0.001	2.594 (1.822–3.695)	< 0.001
7–9	2.280 (1.415–3.672)	0.001	3.297 (1.995–5.448)	< 0.001
≥ 10	4.378 (2.163–8.864)	< 0.001	7.065 (3.38–14.767)	< 0.001
NO*	1.844 (1.521–2.236)	< 0.001	1.458 (1.162–1.830)	0.001
Chemotherapy				
No/unknown	1		1	
Yes	0.842 (0.724–0.979)	0.025	0.721 (0.613–0.848)	< 0.001
Radiotherapy				
No/unknown	1			
Yes	1.036 (0.896–1.198)	0.632		

*HR* hazard ratio, *CI* confidence interval, *SCLC* small cell lung cancer, *LND* lymph node dissected, *LNM* lymph node metastasis, NO^*^, no lymph nodes dissected, *NA* not included in the model due to interference with LNM

### Developing the Prognostic Nomogram Model

**Fig. 1:**
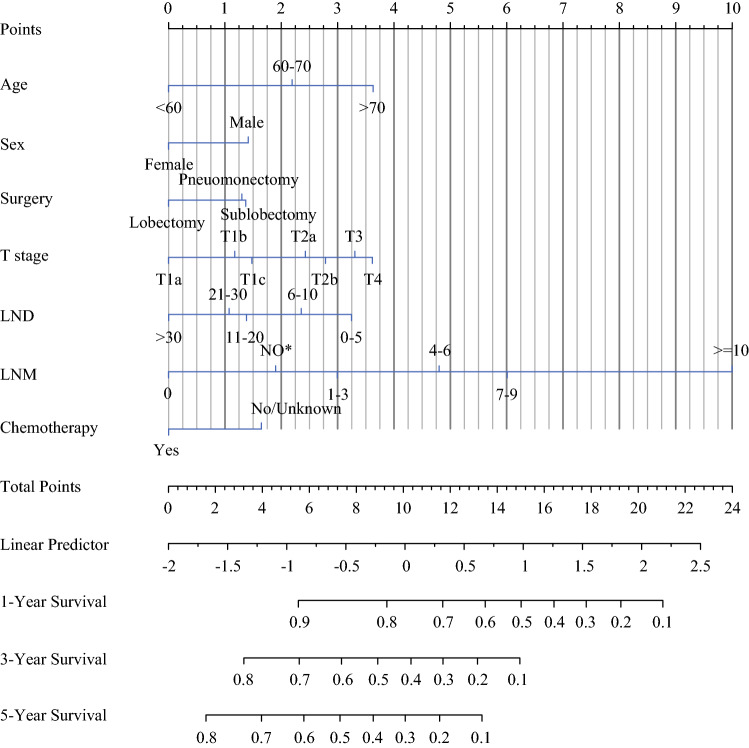
Nomograms for predicting postoperative overall survival in resected limited-stage SCLC patients. *LND* lymph node dissected, *LNM* lymph node metastasis, NO* no lymph nodes dissected, *SCLC* small cell lung cancer

Significant variables of age, sex, surgery, T stage, LND, LNM, and chemotherapy were finally selected for the development of the nomogram model. Each variable was assigned to a point score ranging from 0 to 10 (electronic supplementary Table S1). In the nomogram for OS, LNM showed the largest contribution to prognosis, with a point score of 10, followed by age and T stage (Fig. 1). Notably, sublobectomy and pneumonectomy demonstrated an approximately equal contribution for survival prediction. The individual risk scores were calculated by summing up the score of each variable, and the probabilities of survival at 1, 3, and 5 years were easily determined by locating its corresponding point on the survival scale.

### Model Performance and Validation of the Nomogram

**Fig. 2:**
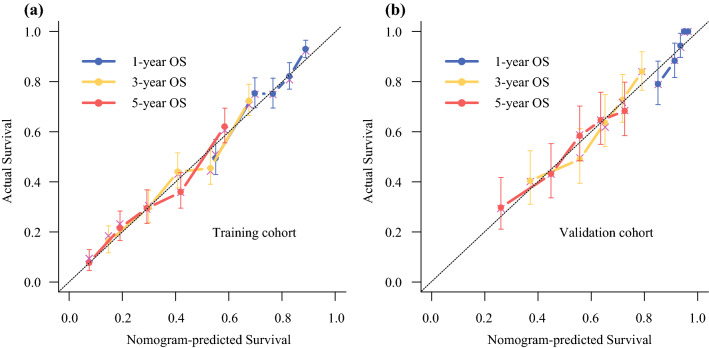
Calibration plots for nomogram-predicted survival (*x*-axis) and actual observed survival (*y*-axis). Calibration curves for OS in the **a** training and **b** validation cohorts; curves for 1-, 3-, and 5-year OS were presented as blue, yellow, and red lines, respectively. Error bars represent 95% confidence intervals. *OS* overall survival

In the training cohort, the C-indexes for the established nomogram were 0.723 (95% confidence interval [CI] 0.685–0.761), 0.722 (95% CI 0.690–0.755), and 0.746 (95% CI 0.710–0.781) for 1-, 3-, and 5-year OS, respectively, and 0.816 (95% CI 0.762–0.870), 0.710 (95% CI 0.658–0.760), and 0.693 (95% CI 0.637–0.750), respectively, in the validation cohort. The calibration plots at 1-, 3-, and 5-year survival showed excellent consistency in the training cohort and acceptable consistency in the validation cohort between the predicted survival probability and actual observation (Fig. 2).

**Fig. 3:**
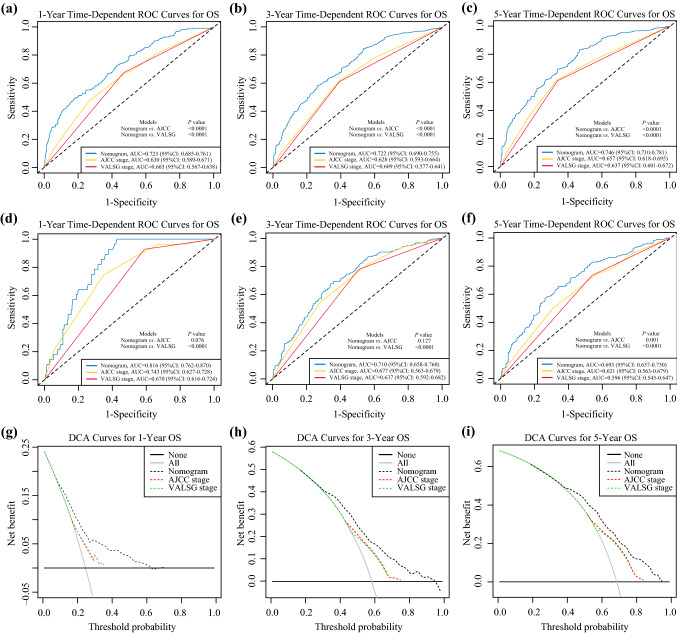
Model performance of the proposed nomogram. **a**–**f** Time-dependent ROC curves of the three prognostic models for predicting 1-, 3-, and 5-year OS. The AUCs of the three prognostic models at each time point of interest were presented and compared in the training and validation cohorts. (g–i) DCA curves of the three prognostic models for 1-, 3-, and 5-year OS. The *x*-axis represents the threshold probabilities and the y-axis measures the net benefit. *ROC* receiver operating characteristic, *AUC* area under the curve, *DCA* decision curve analysis, *OS* overall survival, *AJCC* American Joint Committee on Cancer, *VALSG* Veterans Administration Lung Study Group, *CI* confidence interval

**Fig. 4:**
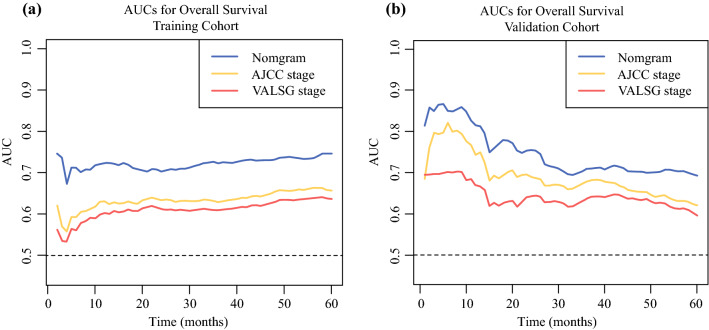
Continuous AUCs of the three prognostic models in the **a** training and **b** validation cohorts throughout the time period of 1–60 months. *AUC* area under the curve, *AJCC* American Joint Committee on Cancer, *VALSG* Veterans Administration Lung Study Group

With regard to prognostic ability, we also conducted comparisons of the model performance between our nomogram and the two conventional staging systems. The 1-, 3-, and 5-year time-dependent ROC curves of the three models are shown in Fig. 3. In the training cohort, all AUCs of the nomogram model were significantly higher than the AJCC (*p* < 0.0001) or VALSG (*p* < 0.0001) staging systems. Similar results were also observed in the validation cohort for comparing this nomogram model with the AJCC or VALSG staging systems, which verified the strong and robust prognostic power of this nomogram. DCA analysis showed that our nomogram model provided significantly increased net benefits over the AJCC or VALSG staging systems within wide and practical ranges of threshold probabilities (Fig. 3), which further verified the better prognostic performance of our nomogram in clinical appliance. Furthermore, we compared the continuous trends of the prognostic performance of each model, and found the AUCs of our nomogram model were higher than that of the AJCC and VALSG staging systems throughout the calculation period (from months 1 to 60), whether in the training or validation cohorts (Fig. 4).

### Risk-Stratifying Ability of the Nomogram

**Fig. 5:**
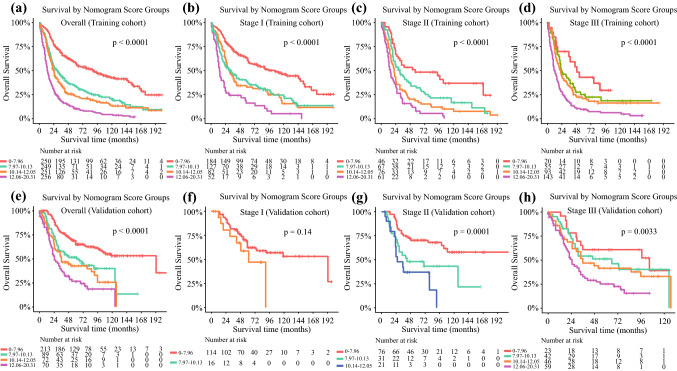
Determinations of risk score groups based on the training cohort and the corresponding survival curves for overall survival in the overall and stage-stratified patients in the **a**–**d** training and **e**–**h** validation cohorts. Subgroups with fewer than 10 patients were omitted from the graphs

Based on the total predictive risk scores, we subcategorized the training cohort into four risk groups, with the optimal cut-off values developed from X-tile software. Detailed subgroups were 0–7.96, 7.97–10.13, 10.14–12.05, and 12.06–20.31 (electronic supplementary Fig. S3). The survival curves for OS showed significant distinctions between any two adjacent groups (*p* < 0.0001) in the training cohort (Fig. 5a) Significant differences were also observed between subgroups when patients were stratified by AJCC stages (*p* < 0.0001) (Fig. 5b–d). This grouping method was then applied to the validation cohort and significant distinctions in survival between different risk groups were also observed, even within certain AJCC staging categories (Fig. 5e–h).

### Webserver Development for the Nomogram

For convenient application of our nomogram, we developed dynamic calculators (electronic supplementary Fig. S4) on the basis of a user-friendly website (https://zengqp1991.shinyapps.io/zengmodel/), which could be used directly by researchers and clinicians. By inputting certain clinical variables, we can easily obtain the corresponding individualized predicted survival probabilities through the output data generated by the website.

## Discussion

Since surgical resection remains an indispensable treatment for early-stage SCLC, and because of the impreciseness of the commonly used AJCC or VALSG staging system for predicting survival for SCLC, a well-developed prognostic model was warranted to compensate for these limitations. In the present study, a novel nomogram prognostic model was established from a large population-based database of limited-stage resected SCLC, and validated using a cohort from our institution. Based on the common clinicopathological variables and treatment information, the individualized probability of survival is readily obtained through our easily accessible online calculator, which could help clinicians in treatment decision making or design of clinical trials. To our knowledge, this was the first attempt to establish a prediction model for the long-term survival of resectable, limited-stage SCLC patients.

Several previous studies have published nomograms regarding survival prediction for SCLC. In 2015, Xie and colleagues developed a nomogram, from a cohort of 938 cases, for predicting OS for SCLC, incorporating peripheral blood markers,^13^ while in 2017, Xiao et al. demonstrated a prognostic nomogram for SCLC patients using a single-institutional cohort of 647 cases.^15^ Regretfully, neither of the two studies applied the more accurate TNM staging system, nor did they assign an independent validation for the model. Recently, Wang et al. developed and validated a web-accessible nomogram for predicting the survival of SCLC patients using the National Cancer Database (NCDB).^16^ Despite the large sample size, this model incorporated the entire stages and treatment patterns, including surgery, chemotherapy, and radiotherapy, which failed to eliminate bias from the interactions between stages and treatment strategies. In our nomogram, we established a surgically based prognostic model and included the limited-stage SCLC cases, which could provide a more accurate probability of survival for this specific subset of patients. The training cohort was obtained from the large and wide geographically distributed SEER database, which guaranteed its generalizability for SCLC patients. Furthermore, this nomogram was validated in an independent cohort of Chinese patients, which increased the universality of this nomogram.

Through univariate and multivariate analysis, age, sex, surgery, *T* stage, LND, LNM, and chemotherapy were recognized as independent prognostic parameters, which was in high accordance with previous reports.^11^^,^^16^^,^^21^^–^23 Notably, radiotherapy was revealed not to be a significant prognostic factor in our study, which may be attributed to the contradictory impact of radiotherapy on patients with different *N* statuses. Wong et al.^12^ reported that radiotherapy deteriorated OS in *N*0 SCLC patients, but improved survival in *N*2-stage patients. As shown in electronic supplementary Table S2, similar effects were also observed in our study. Therefore, to avoid assigning incorrect risk scores to the unspecified patients and to maintain the convenience of this model for clinical use, we did not conduct a subanalysis for prognostic models incorporating radiotherapy. In addition, histology type was not significantly correlated with survival in the univariate analysis, which was contradictory to other studies. In their study, Zhao et al.^24^ reported that combined SCLC was associated with decreased OS compared with pure SCLC. Yokouchi et al.^25^ conducted a retrospective multicenter analysis of 156 resected SCLC patients and revealed no impact of histology type on survival. Given the inconsistency of these studies, we hence excluded histology type from the nomogram construction.

To avoid the overfitting of this nomogram, it was necessary to apply model validation and calibration. In our study, the calibration curve showed optimal accordance between predicted survival probability and actual observation, which revealed good repeatability and reliability of this established model. Furthermore, this nomogram model fits well in the external Chinese validation cohort, which supported the universalized application of this nomogram despite ethnic and geographical differences.

Although the C-indexes of our nomogram failed to reach a high magnitude, this model showed significant higher discriminate ability compared with the AJCC or VALSG staging systems. Additionally, similar superiority was also found in the validation cohort when comparing the nomogram with the other two staging systems. It is notable that when stratifying the validation dataset into several different risk groups using the optimal cut-off values from the training cohort, significant distinctions were also observed in the survival curves, and even the risk groups were further categorized into different AJCC stages, which indicates the satisfied discriminate ability of this nomogram. Admittedly, in stage I patients, the differences were not significant in the survival curves for OS among different risk groups in the validation cohort, and there was also overlapping of survival curves in other stages. The restricted sample size may have contributed to this insignificance.

Based on a convenient scoring system, this prognostic nomogram provides clinicians with better guidance to identify high-risk patients with poor prognosis who may require additional treatment and intensive follow-up. However, our risk score system of different treatment modalities may not be appropriate for direct use, as the decision of treatment strategies involves multiple factors, not merely the TNM stages.

However, several limitations still exist in the present study. First, certain biases may exist due to the nature of this non-randomized, retrospective study. Second, certain weaknesses exist for using the SEER database, which provided only the crude mortality data and lacked in other routinely available parameters (e.g. performance score, smoking status, pulmonary functions, or body mass index) or any of the essential comorbidities (e.g. pulmonary hypertension, congestive heart failure, vascular disease, or renal failure). Dependency on the SEER database prevented us from including these parameters in this model. Moreover, the sequence of chemotherapy or radiotherapy with surgery was not considered, as the exact time for treatment was lacking in the SEER database. Consequently, we assumed chemotherapy or radiotherapy to be baseline variables instead of time-varying covariates; however, this assumption undoubtedly ignored the effects of treatment sequence on patients’ survival. Hence, we will be conducting further multicenter research that incorporates relative completed clinicopathological variables, as well as detailed information regarding additional treatment, to refine the predictive power and generalizability of our model. It is hopeful that our nomogram model will create a more precise survival prediction when incorporating those unanalyzed parameters, which may include performance score, smoking status, pulmonary function, body mass index, and detailed additional treatment, as well as the above-mentioned comorbidities.

## Conclusions

To date, no well-established tool has been reported for the prediction of survival in resected limited-stage SCLC patients. Our nomogram model was developed from integrated prognostic variables using a population-based US database, and externally validated in a Chinese cohort. This model consistently achieved appreciable prognostic ability, reliability, and clinical applicability, and hence may offer clinicians instructions for survival counseling, treatment strategy making, and clinical trial design. Furthermore, this proposed nomogram was also deployed into a website server for convenient application.

## Supplementary Information

Below is the link to the electronic supplementary material.

Supplementary material 1 (DOCX 1095 kb)
