# Supplementary material for: Development and Validation of a Nomogram Prognostic Model for Resected Limited-Stage Small Cell Lung Cancer Patients
Source: Ann Surg Oncol. 2021 Mar 2;28(9):4893–904. doi: 10.1245/s10434-020-09552-w (PMC8349336; doi:10.1245/s10434-020-09552-w)

**Supplementary Table S1**. **Point assignment of prognostic scores**

| **Variable** | **Prognostic Score** |
| --- | --- |
| Age, y  < 60  60-70  > 70 | 0  2.190  3.631 |
| Sex  Female  Male | 0  1.414 |
| Surgery  Lobectomy  Sublobectomy  Pneumonectomy | 0  1.372  1.298 |
| T stage  T1a  T1b  T1c  T2a  T2b  T3  T4 | 0  1.174  1.479  2.432  2.781  3.306  3.618 |
| LND  0-5  6-10  11-20  21-30  > 30 | 3.244  2.355  1.383  1.073  0 |
| LNM  0  1-3  4-6  7-9  >= 10  NO* | 0  2.994  4.800  6.006  10.000  1.898 |
| Chemotherapy  No/Unknown  Yes | 1.647  0 |

LND, lymph node dissected; LNM, lymph node metastasis; NO^*^, no lymph node dissected.

**Supplementary Table S2. Univariate analysis for patients with N0, N1, and N2 disease in the training and validation cohort.**

|  | Training cohort | | | | | | Validation cohort | | | | | |
| --- | --- | --- | --- | --- | --- | --- | --- | --- | --- | --- | --- | --- |
|  | **N0** | | **N1** | | **N2** | | **N0** | | **N1** | | **N2** | |
| Variables | **HR**  **(95% CI)** | ***P* value** | **HR**  **(95% CI)** | ***P* value** | **HR**  **(95% CI)** | ***P***  **value** | **HR**  **(95% CI)** | ***P* value** | **HR**  **(95% CI)** | ***P* value** | **HR**  **(95% CI)** | ***P***  **Value** |
| Chemotherapy  (Yes *vs*. No/Unknown) | 0.789  (0.646-0.963) | 0.020 | 0.760  (0.528-1.096) | 0.142 | 0.441  (0.312-0.624) | < 0.001 | 0.551 (0.238-1.276) | 0.164 | 0.666 (0.287-1.544) | 0.344 | 0.151 (0.070-0.327) | < 0.001 |
| Radiotherapy  (Yes *vs*. No/Unknown) | 0.990  (0.792-1.239) | 0.932 | 0.768  (0.569-1.037) | 0.085 | 0.675  (0.501-0.908） | 0.009 | 2.418 (1.288-4.539) | 0.006 | 0.992 (0.586-1.680) | 0.975 | 0.558 (0.361-0.861） | 0.008 |

**Supplementary Figure S1**. Flow chart for enrollment of the SEER database
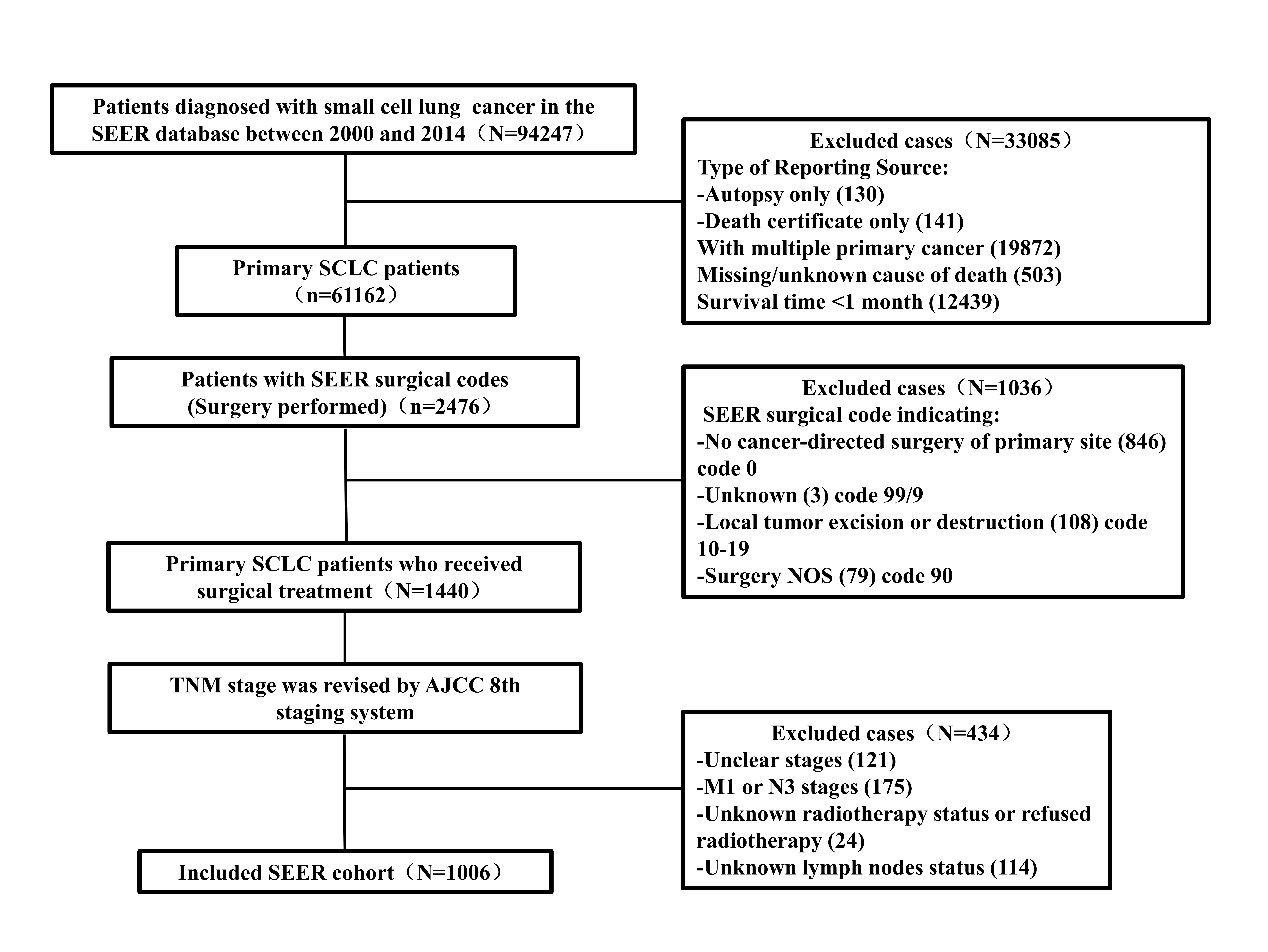


SCLC, small cell lung cancer; NOS, not otherwise specified.

**Supplementary Figure S2**. Kaplan-Meier survival curves in the univariate analysis.


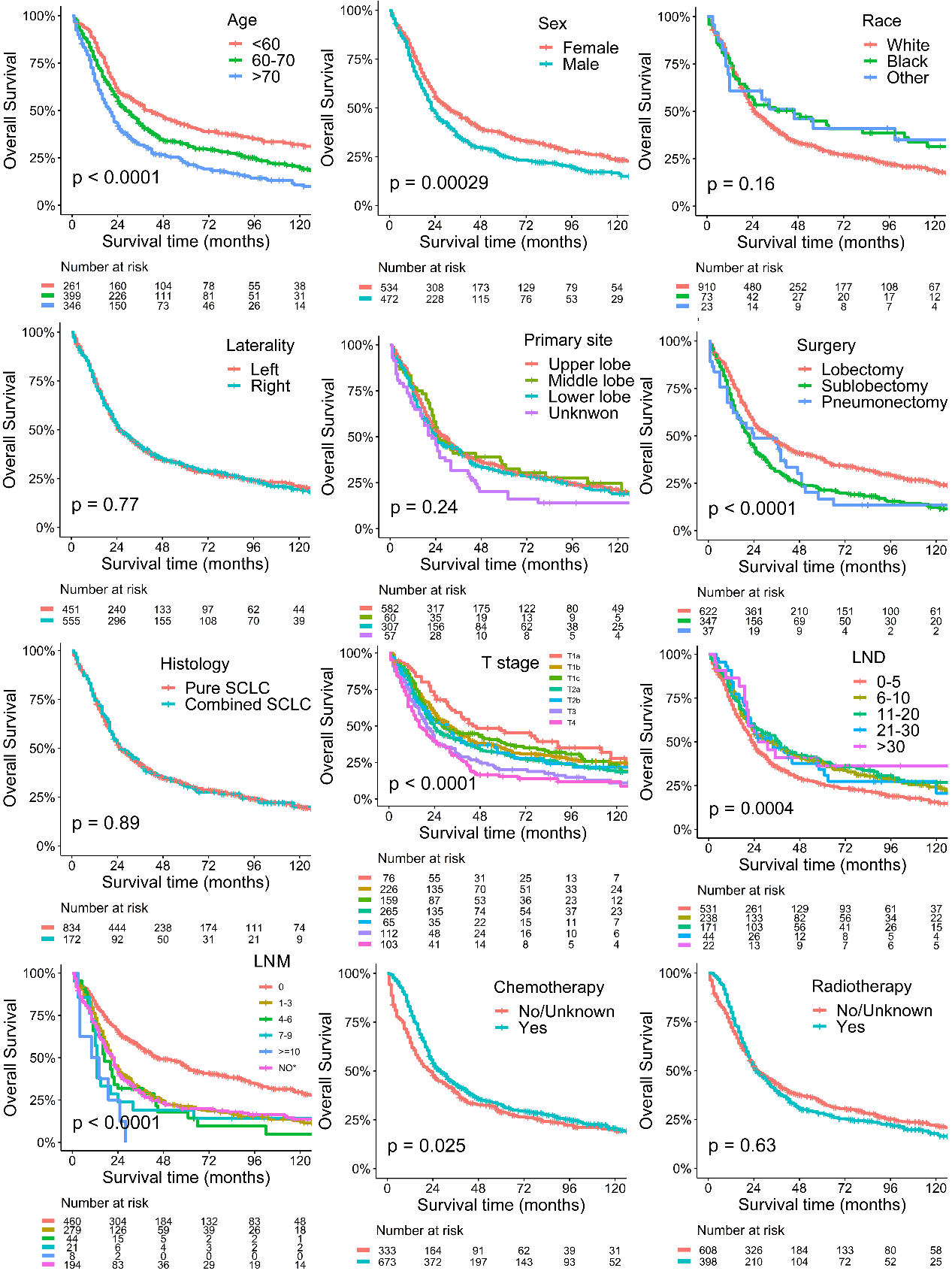


SCLC, small cell lung cancer; LND, lymph node dissected; LNM, lymph node metastasis; NO*, no lymph node dissected.

**Supplementary Figure S3**. The optimal cutoff values for risk scores determined by X-tile software (Yale University, New Haven, CT, USA) in patients of the training cohort.
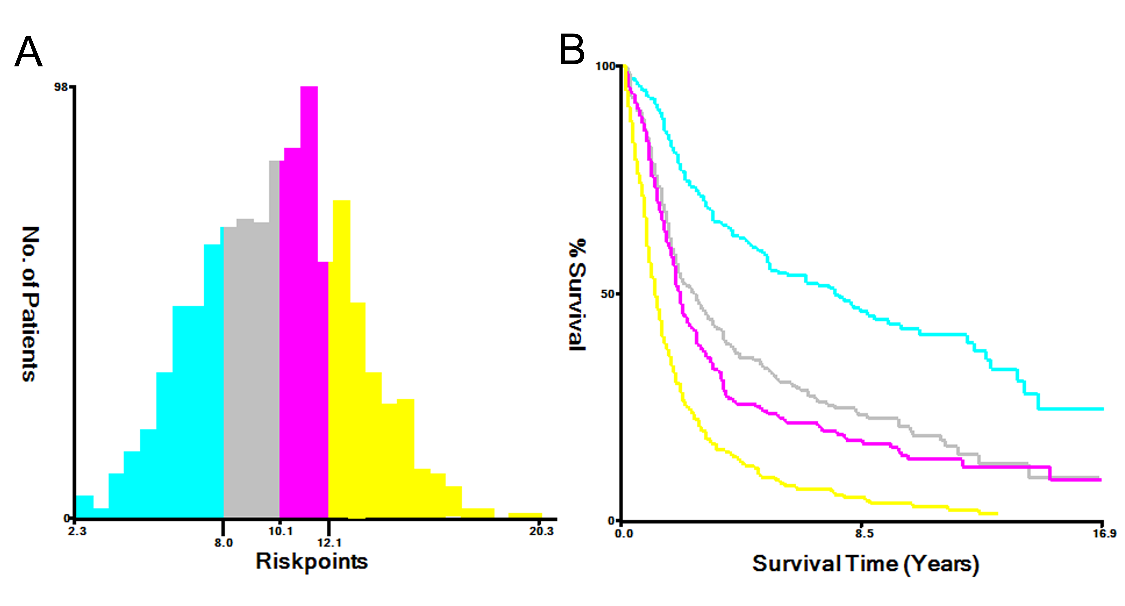


Risk score groups were developed using the primary outcome of OS.

**Supplementary Figure S4**. Online calculator interface for our nomogram. The left side showed this input field and run button. The right side showed the corresponding output figure and table (A, survival curve; B and C, estimated survival probability and 95% confidence interval).


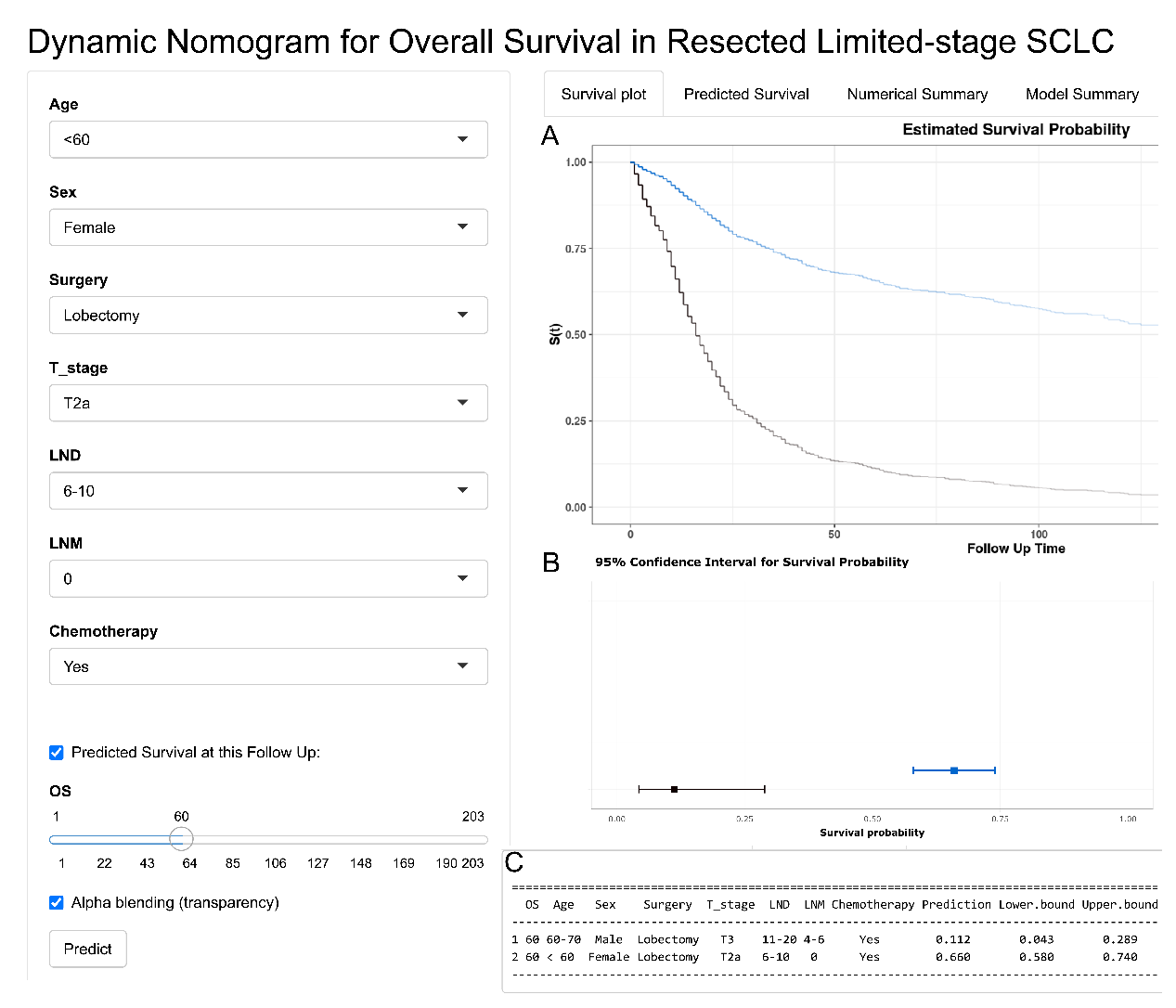

Supplement: Supplementary file 1 — Supplementary material 1 (DOCX 1095 kb) [file 10434_2020_9552_MOESM1_ESM.docx]
